# Impact of a multimedia website with patient experiences of multiple sclerosis (PExMS) on immunotherapy decision-making: study protocol for a pilot randomised controlled trial in a mixed-methods design

**DOI:** 10.1186/s40814-020-00749-0

**Published:** 2021-01-07

**Authors:** Anna Barabasch, Karin Riemann-Lorenz, Christopher Kofahl, Jutta Scheiderbauer, Desiree Eklund, Ingo Kleiter, Jürgen Kasper, Sascha Köpke, Susanne Lezius, Antonia Zapf, Anne Christin Rahn, Christoph Heesen

**Affiliations:** 1 Institute of Neuroimmunology and Multiple Sclerosis (INIMS), University Medical Center Hamburg-Eppendorf (UKE), Hamburg, Germany; 2 Institute of Medical Sociology, University Medical Center Hamburg-Eppendorf (UKE), Hamburg, Germany; 3 Patient representative, Hamburg, Trier, Germany; 4 Marianne-Strauß-Klinik, Behandlungszentrum Kempfenhausen für Multiple Sklerose Kranke gGmbH, Berg, Germany; 5 Department of Nursing and Health Promotion, OsloMET, Oslo Metropolitan University, Oslo, Norway; 6 Institute for Clinical Nursing Science, University of Cologne, Cologne, Germany; 7 Institute of Medical Biometry and Epidemiology, University Medical Center Hamburg-Eppendorf (UKE), Hamburg, Germany; 8 Department of Health Services Research, Carl von Ossietzky University of Oldenburg, Oldenburg, Germany; 9 Department of Neurology, University Medical Center Hamburg-Eppendorf (UKE), Hamburg, Germany

**Keywords:** Multiple sclerosis, Decision-making, Decision support, Web-based experiential information, Patient experiences, Narrative information

## Abstract

**Background:**

A variety of management options (e.g. immunotherapies, lifestyle interventions, and rehabilitation) are available for people with relapsing-remitting multiple sclerosis (RRMS). Besides coping with the diagnosis, people with MS (pwMS) have to make complex decisions such as deciding about immunotherapies. In addition to factual information, reports of patient experiences (PEx) may support patients in decision-making. The added value of PEx in decision-making is not clear, and controlled studies are rare. Therefore, systematic methods are necessary to develop and analyse PEx. As there are no evaluated PEx for MS in Germany, we are currently creating a website presenting PEx structured according to topics and illustrated by video, audio, and text files. We aim to determine the feasibility of an intervention using PEx and evaluate whether PEx may help pwMS in their immunotherapy decision-making processes as a supplement to evidence-based information.

**Methods:**

This project will follow the Medical Research Council framework for development and evaluation of complex interventions. After the development of a website with PEx, a randomised controlled pilot trial (pilot RCT) will be conducted in 2–3 MS centres, clinics, or rehabilitation centres including 55 pwMS and accompanied by a process evaluation. Patients with a RRMS diagnosis considering immunotherapy are eligible. The primary outcome is decision self-efficacy. Secondary outcomes include preparation for decision-making, decisional conflict, risk knowledge, confidence in active participation, affective forecasting, social support, and self-reported impact of eHealth on its users. Participants will be randomly assigned either to (i) an intervention group with 4 weeks access to an evidence-based patient information resource and the PExMS-website as an adjunct or to (ii) the control group with access to evidence-based information alone. A 6-member advisory panel involving representatives of pwMS, researchers, and neurologists, who accompany the whole project, will mentor this pilot RCT.

**Discussion:**

The intervention was developed with systematic methods, created with active patient involvement and in critical appraisal by an expert advisory panel. The study is innovative as it contributes to the controversial evidence on the use of PEx in the context of evidence-based patient information.

**Trial registration:**

ClinicalTrials.gov, NCT04236544

**Supplementary Information:**

The online version contains supplementary material available at 10.1186/s40814-020-00749-0.

## Background

Multiple sclerosis (MS) is a chronic, degenerative disease of the central nervous system, which is causing remitting and progressive physical and psychological dysfunctions. Three major disease course forms of MS can be separated: relapsing-remitting MS (RRMS) (the most frequent type), secondary progressive MS (SPMS), and primary progressive MS (PPMS). Many people with MS (pwMS) are diagnosed at age 20 to 40, mostly with RRMS [1, 2]. In Germany, about 200,000 cases are prevalent [3].

For RRMS, 18 disease-modifying therapies are available, but those are only partially effective. Most of them have substantial side effects, in the worst-case life-threatening. The long-term effectiveness remains unclear. Moreover, other approaches such as lifestyle interventions, rehabilitation, and so-called complementary alternative therapies might be helpful [4, 5]. This makes the decision-making process for such treatments complex, while at the same time, the information needs of pwMS are often not met [6–8].

Information provision with carefully developed decision aids including group education [9] and face-to-face coaching [10] bears positive outcomes and is urgently needed but not everywhere available. Among chronically ill people, pwMS are among the most frequent internet users being confronted by an overwhelming amount of web-based information of variable quality and aims [11–13].

When faced with new health concerns or treatments, patients search not only for fact-based information but also seek other patient experiences (PEx) [14]. There is limited but increasing research looking at the value of PEx [14–24]. The design and quality of these studies including PEx as an intervention varies [18, 24]. Also, there is no agreed-upon operationalisation of PEx, which are also called narratives, exemplars, anecdotes, testimonials, or case histories [24]. This study will use the term PEx, specifically to denote online messages of patients in first-person as text, audio, or video files.

Little is known on the outcomes of PEx, especially how they influence decision-making [18, 24]. PEx have the potential to enhance the motivation in engaging with patient decision aids and the perceived experience of using it, especially in a population with lower health literacy [18]. According to cognitive learning theories, experiential information allows people to process, store, and save information more effectively than didactic information because it is vivid [17, 23, 25]. Moreover, patients have trouble forecasting feelings, especially their intensity and duration, which results in inaccurate affective forecasts [26]. PEx may increase the perceived ability to make accurate affective forecasts, which is the peoples’ prediction about how they will feel during future or imagined events. People used to focus on losses rather than continuity or even potential gain. Also, they fail to envision how their coping skills will help to manage the new situation and develop adaptation, which is important in decision-making processes [27]. Through PEx, patients may gain feelings of control and confidence facilitating self-management and coping uncertainty [16, 17, 23, 25, 28].

The internet provides a wide range of opportunities to share and learn about other PEx via blogs and discussion groups. Different motivations and mostly negative or dramatic experiences within the health system drive contributors to social media platforms, which lead to a selective picture of the disease, treatments, and health care resources. More numerous but inconspicuous PEx remain unnoticed. Beyond the huge amount of free-floating information on the web, concerns exist about the use of experiential information because of considerable risks of selectivity and biased information even if presented along with balanced information [25]. Looking at poor existing evidence of PEx, while not differentiating between varying kinds, the German ‘Guideline Evidence-based Health Information’ does not recommend narratives being part of evidence-based health information [29].

However, the high usage of social media strongly underlines pwMS’ needs of PEx. Therefore, there is an unmet need for care-oriented research on making PEx accessible and studying their impact. To our knowledge, there are no evaluated PEx in MS in Germany so far. This pilot randomised controlled trial (pilot RCT) will be the first testing of an intervention including PEx of MS (PExMS) as video and audio files, gained through a systematic qualitative research approach.

## Objectives

By embedding a process evaluation within the pilot trial, the primary study objective is to test the feasibility of an intervention using PEx as a supplement to evidence-based information to establish whether it is possible to carry out a large future RCT. The specific objectives are to determine the following:

1. Acceptability of recruitment, randomisation, and consent procedure
2. Acceptability and feasibility of collecting reliable and valid data on primary and secondary outcomes
3. Acceptability of the intervention and processes to participants and clinical staff
4. Suitability of the intervention used
5. Barriers to adherence
6. Explore aspects of the trial design and conduct with a patient and public involvement group

We hypothesise that PEx may help pwMS in the decision-making process for or against immunotherapy or their different forms, respectively. Therefore, the secondary objectives are (1) to obtain pilot data on the likely difference between the intervention and evidence-based information alone regarding decision self-efficacy; (2) to inform the sample size calculation for the substantive randomised clinical trial; and (3) to control for potential bias caused by PExMS.

## Methods

### Trial design

The PExMS project follows the guidance of the Medical Research Council [30] for the development and evaluation of complex interventions and adheres to the SPIRIT checklist (see Additional file 1). The process has a development as well as a feasibility and piloting phase.

1. Development: The PExMS website was developed within a qualitative interview study, where PEx were collected (see ‘Intervention description’).
2. Feasibility and piloting: First, people with RRMS (see ‘Intervention description’) will be asked about their experiences with the PExMS website (e.g. usability, acceptance, layout, navigation) and the outcome measurement procedures.
  Second, in a two-arm pilot RCT, we will compare a combination of the PExMS website and evidence-based patient information with evidence-based patient information alone. The pilot trial is intended to test recruitment, outcome measures, data collection (sampling and timing), randomisation procedures, and acceptability of the intervention for pwMS and practitioners, and gain exploratory results that could be used for sample size estimations in future RCTs. We want to assess whether the intervention was conducted as intended (fidelity) and assess the quantity of the implemented intervention (dose).
  A mixed-methods process evaluation will be applied. In the recruitment phase, we will perform semi-structured telephone interviews with all responsible persons in participating centres to assess perceptions of strengths, weaknesses, risks, and opportunities of the study.
  After completing the study, centres which succeeded in recruitment and centres that dropped out prematurely or failed in recruitment will be interviewed and will be asked to fill out a process evaluation questionnaire to assess acceptability of the recruitment, randomisation and consent procedure, and the intervention itself.
  Additionally, approximately 10 patients, namely maximum users and minimum users, of the PExMS-website and those having dropped out will be interviewed in each group and will be requested to complete a process evaluation questionnaire.

### Study setting

The study will be conducted in different participating neurological practices, clinics, or rehabilitation centres located in Germany.

### Eligibility criteria

Eligibility criteria are RRMS, being ≥ 18 years old, and considering immunotherapy for mild/moderate or (highly) active courses. Participants will be excluded if they are diagnosed with SPMS or PPMS, have major cognitive deficits, and/or poor German language skills. Also, prior participation in the development phase of the PExMS website is an exclusion criterion.

## Interventions

### Explanation for the choice of comparators

The control group will receive access to the DECIMS (Decision coaching in MS)-Wiki (www.wiki2.kkn-ms.de), an evidence-based patient information website focusing on MS immunotherapies for 4 weeks. DECIMS-Wiki is considered to provide excellent factual information with figures, but without PEx and is regarded as the gold standard. Further information is described elsewhere [31]. Site visits will be recorded and tracked with the explicit consent of the participants. At the end of the 4-week period, study participants will be invited to complete follow-up questionnaires.

### Intervention description

#### Development of an intervention website with PEx

**Fig. 1:**
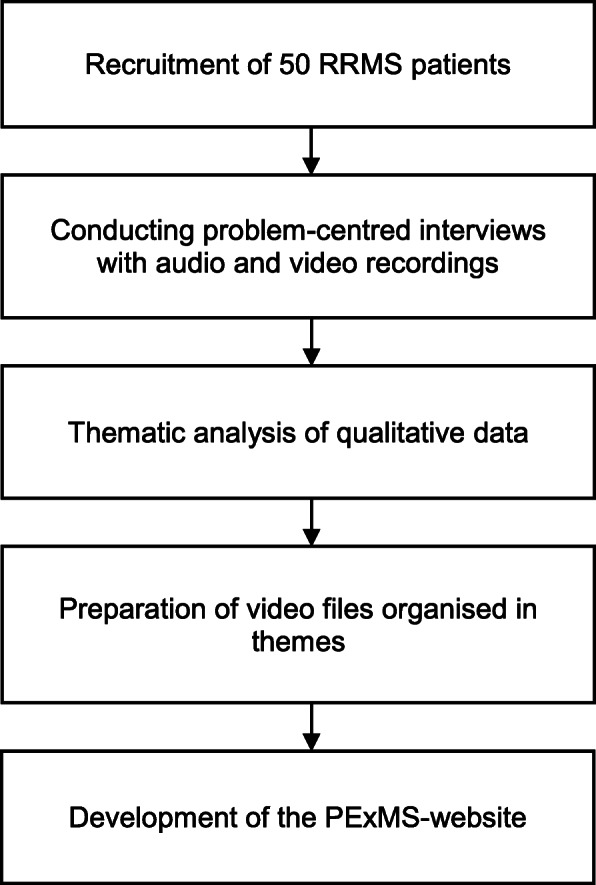
Flow diagram showing the process of developing a website with PEx

The intervention is a multimedia PExMS website providing short videos, audio recordings, and written excerpts of 50 pwMS. The development of the intervention followed largely recommendations for standardised qualitative research provided by international DIPEx (Database of Individual Patients’ Experience of illness) association. This collaboration of researchers and health professionals use standardised qualitative research methods to understand PEx and tries to provide ‘balanced’ information from original interview data. However, this project is not affiliated to DIPEx. Figure 1 shows the process of the website’s development.

Data for the website was generated in a qualitative interview study with RRMS patients (≥ 18 years old) across Germany. A maximum variation sampling strategy [32] was used aiming to cover experiences with all licensed MS drug treatments. A problem-centred interview guide according to Witzel [33] was applied involving open-ended and closed questions on PEx with diagnosis, talking about MS diagnosis with others, MS in everyday life, and experiences with management approaches (DMT, lifestyle intervention, alternative medicine, rehabilitation) as well as decision-making processes. Interviews were audio- and videotaped, and afterward transcribed. We analysed qualitative data thematically [34] using deductive and inductive categories. All themes representing experiences with MS in everyday life and with therapies were organised in sub-themes and illustrated by text, audio, and video clips to provide the basis for the PExMS-website.

Feasibility testing of the PExMS website will be conducted with people with RRMS (≥ 18 years old) not previously involved in the development phase of the PExMS website. They will receive access to the PExMS website for 1 week for evaluation of the preliminary version of the website. We will record log information (e.g. date, number of logins, and file name of the webpage) by the webserver to assess users’ navigation on the website. Then, two focus group discussions with 5–8 RRMS patients per group will be conducted asking for experiences with the website (e.g. usability, acceptance, layout, navigation) and suggestions for improvement [19]. The focus groups will be audiotaped and transcribed verbatim. Moreover, outcome measures, which are described in the following, will be tested. We will analyse this data descriptively using SPSS Statistics™ Version 25 and use the results for the optimisation of the content and presentation of PEx on the website. The final tool will be used as an intervention in the pilot trial.

#### The intervention group

The intervention group will receive access to the PExMS website and the DECIMS-Wiki for 4 weeks. As in the control group, the site visits are tracked. At the end of the 4 weeks, pwMS will be invited to complete follow-up questionnaires.

### Criteria for discontinuing or modifying allocated interventions

Studies investigating PEx as a patient information tool found no adverse events [14, 22, 35]. Therefore, we do not expect any relevant harm of the intervention and did not establish criteria for discontinuing or modifying the allocated intervention for participants. Nevertheless, study participants may withdraw their participation at any time upon request.

### Strategies to improve adherence to interventions

All participating neurological practices, clinics, or rehabilitation centres will receive a study folder including all relevant documents. Also, regular phone calls and telephone conferences will be conducted for arising questions and updates and for sharing experiences.

Usage of the intervention will be monitored by tracking the site visits. PwMS will be contacted by regular email reminders by a member of the coordinating centre to use the intervention. In case of non-use, participants will be reminded by email or telephone.

### Outcomes

#### Primary outcomes

Primary outcomes will be assessed by mixed-methods consisting of interviews and process evaluation questionnaires, which include the following overall feasibility and process-related measures:

1. Number of participants consented, recruited, randomised, withdrawn, and retained
2. Numbers with completed outcome measures or lost to follow-up
3. Acceptability of the intervention in terms of frequency and duration of usage as well as experience, motivation, barriers, and facilitators to use the intervention—from the pwMS and participating study centre perspective
4. Acceptability of the study procedures in terms of barriers and facilitators to implementation—from the pwMS and participating study centre. We will also ascertain the number and type of adaptations made to implementation strategies including information on how and why.

#### Secondary outcomes

We hypothesise that PEx will lead to a positive effect on decision self-efficacy [23]. This will be assessed by the 11-item ‘Decision Self-Efficacy Scale’ (DSES) [36], which measures self-confidence in decision-making on a five-point-Likert scale. It ranges between 0 (not at all confident) and 4 (very confident). For the total score, items are summed, divided by 11, and multiplied by 25. A total score of 0 means ‘extremely low self-efficacy’, and a score of 100 means ‘extremely high self-efficacy’. The Decision Self-Efficacy Scale shows adequate internal consistency achieving a Cronbach’s alpha of 0.84 [37].

To assess how useful a decision support intervention is in preparing pwMS to communicate at a consultation visit and to make a decision concerning a therapy, the 10-item ‘Preparation for Decision Making’ (PrepDM) [30] scale will be used. It has a five-point-Likert scale format and is designed to be administered after the consultation visit to discuss treatment options. Higher scores show a higher perceived level of preparation for decision-making. Cronbach’s alpha ranges from 0.92 to 0.96 [38].

It is assumed that patients seeing PEx develop greater MS risk knowledge, which we will assess with a short version of the 19-item ‘Risk Knowledge in Relapsing Multiple Sclerosis 2.0’ (RIKNO) [39, 40]. Item difficulty is high ranging from 0.07 to 0.79. RIKNO 2.0 has good internal consistency achieving a Cronbach’s alpha of 0.73 [40].

‘Informed choice’ [41] will be assessed by the Multidimensional Measure of Informed Choice (MMIC) as in previous trials [9, 42]. It comprises three dichotomous dimensions: risk knowledge (good, poor), attitude towards or against immunotherapy or change of immunotherapy (positive, negative), and choice (uptake or non-uptake of the intervention under consideration) leading to eight types of choices. PExMS is expected not to change the percentage of informed choices. Besides, the distribution of decisions made for or against immunotherapy is expected to remain unchanged [22, 35].

The 4-item ‘SURE’ (Sure of myself, Understand information, Risk-benefit ratio, Encouragement) scale [43] addresses decisional conflict in patients. It will assess patients’ perception of uncertainty about decision making for a therapy. The reliability of SURE with a Cronbach’s alpha of 0.54 is moderate [43].

We will assess patients’ preferences for involvement in treatment decisions by the 5-item Control Preferences Scale (CPS) [44]. In a validation study, a Cronbach’s alpha of 0.72 was attained [45].

PEx may give pwMS confidence in active participation and management in medical care [46]. Therefore, the 13-item ‘Patient Activation Measure 13’ (PAM13-D) [47] will be used which is a reliable and valid measure of patient activation with a four-point-Likert scale. It shows good internal consistency achieving a Cronbach’s alpha of 0.87 [47].

The eHealth Impact Questionnaire ‘eHIQ’ [46, 48] measures attitudes of users towards a website which they recently viewed. It is divided into the 11-item eHIQ-Part 1 and the 26-item eHIQ-Part 2. The former represents general attitudes towards using the internet to access health information. The second part is related to the effects of using a specific health-related website on three subscales: (1) confidence and identification, (2) information and presentation, and (3) understanding and motivation. Both answering formats range from 1 ‘strongly disagree’ to 5 ‘strongly agree’. The eHIQ shows good internal consistency achieving a Cronbach’s alpha of ≥ 0.77. A German translation will be validated and used. Moreover, identifying with others who show their experiences on a website, knowing that other pwMS are handling similar problems and learning how they manage difficult issues can reduce the feeling of isolation and improve the sense of social support and group membership [14, 25]. Reformulated items from the subscale ‘confidence and identification’ of the eHIQ will be used to assess social support.

‘Affective forecasting’ is defined as the patient’s perceived ability to make predictions of future feelings about difficult health procedures or disabling health conditions. We hypothesise that PEx could have an impact on patients’ ‘affective forecasting’ [23]. As no validated questionnaire exists for this, a preliminary scale with 5 to 10 items will be developed through a review of relevant literature. We will conduct cognitive interviewing using the think-aloud methodology after showing approximately five patients clips from the website with PEx and the preliminary scale. The clips will show how other patients have coped with emotions during a certain situation as well as the intensity and duration of an emotional state, which the patients experienced. Pilot testing of the revised scale will be conducted applying again cognitive interviewing for face and content validity.

### Demographic/clinical variables/covariates

As a control parameter, the ‘Hospital Anxiety and Depression Scale’ (HADS) will be administered exploring symptoms of anxiety and depression [49]. We will also measure patient-reported disability of pwMS using the ‘Patient Determined Disease Steps’ (PDDS) [50]. The ‘Stage of Decision Making’ [51] questionnaire asks for the patient’s readiness to engage in decision-making, progress in decision-making, and susceptibility to considering or re-considering options. It may be useful in screening out pwMS who may not benefit from decision aid interventions.

For a list of outcomes and a schedule of assessments, see Table 1.

**Table 1:** Study assessment

	Study period			
	Enrolment	Pre-study baseline/allocation	Study/post-allocation	
**Timepoint**	***−t***_***1***_	**0**	***t***_***1***_	***t***_***2***_
**Enrolment:**				
**Eligibility screen**	X			
**Informed consent**	X			
**Randomisation**		X		
**Sociodemographic data and MS-related data**		X		
**Assessments**				
**PDDS**		X		
**HADS**		X	X	
**DSES**		X	X	
**PrepDM**			X	
**RIKNO**		X	X	
**MMIC**		X	X	
**SURE**		X	X	
**CPS**		X	X	
**PAM 13-D**		X	X	
**Affective forecasting**		X	X	
**Social support**			X	
**Stage of decision making**		X		
**eHIQ-Part 1**		X		
**eHIQ-Part 2**			X	
**Patient treatment decision/Uptake**				X

*t1* after 4 weeks usage of intervention or comparator website(s), *t2* after physician encounter

### Participant timeline

**Fig. 2:**
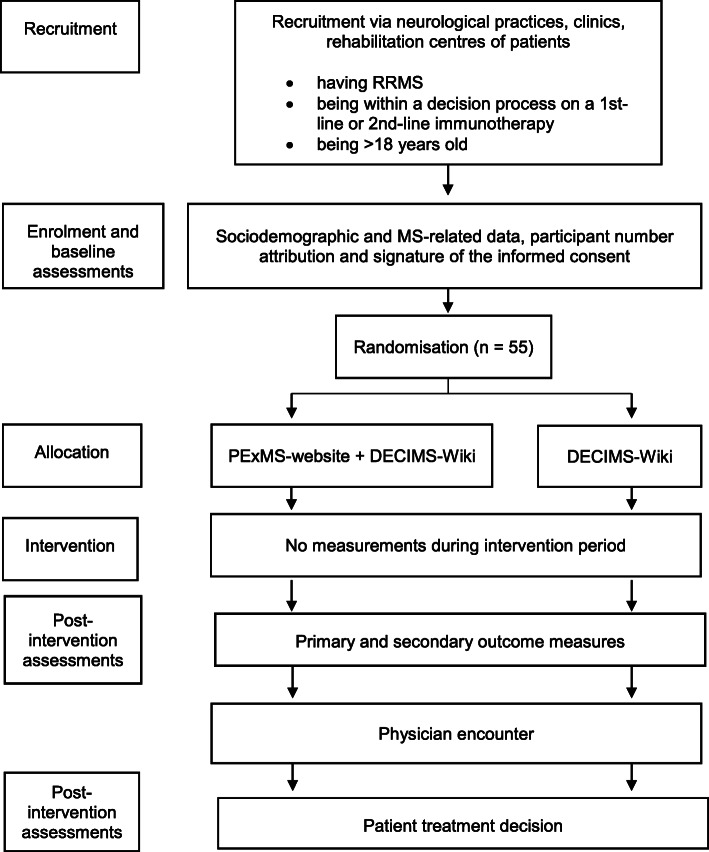
Study flowchart

Figure 2 shows the flow of enrolment, interventions, assessments, and visits for pwMS.

### Sample size

To our knowledge, there is no previous research using decision aids with patient narratives and addressing decision self-efficacy. However, since the main aim of the pilot RCT is not to give a formal assessment of efficacy, but rather to evaluate trial procedures and processes as well as to get estimates of parameters for the main RCT sample, the sample size justification is based on rules of thumb for pilot RCTs as given by Sim and Lewis. The authors recommend for a two-armed pilot RCT a total sample size of 55, which would minimise the overall sample size for the main RCT for small to medium standardised effect sizes [52–54]. Additionally, pilot RCTs on decision support tools in MS showed comparable sample sizes [31, 55].

### Recruitment

Participants will be recruited by 2–3 neurological practices, clinics, or rehabilitation centres in Germany. Potential participants will be invited to read the patient information sheet before deciding whether to take part in the study. Informed consent will be obtained by a physician or trained scientific personal in the participating neurological practices, clinics, or rehabilitation centres.

Once consent has been obtained, participants will get a link and an individual access key to the study registration webpage, which is QuestBack Unipark (2019), an online survey tool. There, participants will be asked for socio-demographic and MS-related data.

## Assignment of interventions

### Sequence generation

PwMS will be randomly allocated in blocks with variable block length at a 1:1 ratio to the intervention or the control group. The concealed allocation sequence will be computer-generated by the randomisation sequence of QuestBack Unipark.

### Concealment mechanism

The randomisation will be automatically performed via QuestBack Unipark and will be therefore concealed.

### Implementation

PwMS will be enrolled in the participating neurological practices, clinics, or rehabilitation centres. The allocation sequence and participants’ assignment to interventions will be automatically generated by QuestBack Unipark.

### Blinding

Blinding of the pwMS is pursued but is only possible to a limited extent due to the type of intervention. We will explain in our information and consent form that we intend to assess whether pwMS find different health information websites helpful on a range of self-reported outcomes. Researchers involved in study recruitment, administration of interventions, and data analysis will be blind to the allocation of intervention and control group. Since we do not expect relevant adverse events, no procedure for unblinding is planned.

## Data collection and management

### Plans for assessment and collection of outcomes

Data from the Pilot RCT will be collected at four time points using web-based questionnaires from QuestBack Unipark (see ‘Outcomes’). This promotes the quality of data due to the avoidance of data entry and coding errors by third persons, reduced administrative burden, prevention of item nonresponse, and automatic application of skip patterns. Patients’ treatment decisions will be assessed after the intervention by a physician of the participating neurological practices, clinics, or rehabilitation centres and documented by physicians or study nurses of the study centre.

### Plans to promote participant retention and complete follow-up

PwMS will be contacted via regular email reminders by a member of the coordinating centre when it is time to fill in the questionnaires and will be asked to complete these within a specified period. Those who miss the completion will be reminded by email or telephone. PwMS who withdraw from the study will be requested whether they agree to continue to complete the questionnaires related to the primary and secondary outcomes.

### Data management

PwMS will enter their data in QuestBack Unipark. When the data collection is complete, it will be exported from QuestBack Unipark. The variables will be then renamed using a previously prepared codebook, and initial data checks will be conducted in IBM SPSS Statistics 25 by a statistician of the coordinating study centre. Pseudonymised data will be stored at the Institute of Neuroimmunology and Multiple Sclerosis at the University Medical Center Hamburg-Eppendorf. The analysis and usage of the data by the study principal investigator and his staff is done in pseudonymised form. The data collected during the study will only be made available for the scientific community in anonymised form. The same applies to the publication of the study results.

### Confidentiality

All data collected during the study will be kept strictly confidential. All electronic data from the pilot RCT will be pseudonymised and there will be no possibility to link data to persons without access to the code list. Through the personal access of patients to the PExMS website or DECIMS-Wiki based on a personal account (sent via email), it will be possible to individually track the use of the websites (e.g. frequency, use of different parts), which will be used to analyse user behaviour and the feasibility of the study design. User’s IP address will be stored by the system but shielded to anyone but the system administration. A personal account name will be used to connect users and pseudonyms.

## Analysis

The feasibility and process evaluation findings will inform the design of a future fully powered, randomised controlled trial to formally evaluate the effectiveness of the intervention. Therefore, we seek a drop-out rate < 20% and to collect > 60% of patient-reported outcomes (questionnaire response rate). Frequencies will be determined for categorical variables, and summary statistics (mean and standard deviation or median and quartiles, as appropriate) will be calculated for quantitative variables. Exploratory formal hypothesis testing using ANCOVA models will be performed since this is a pilot RCT. Estimated differences in outcome measures of efficacy adjusted for baseline values will be calculated. No interim analyses and stopping guidelines are planned. Initially, all analyses will be performed on complete cases. If necessary, sensitivity analyses will be conducted with imputed datasets derived by multiple imputation or mean value imputation.

Qualitative data gained through interviews will be managed using MAXQDA Analytics Pro 2018 and analysed thematically following Braun and Clarke [34].

## Oversight and monitoring

### Composition of the coordinating centre and trial steering committee

The project is mentored by an advisory panel of seven persons, which includes representatives of MS patients, researchers, and neurologists. It is a key component of the DIPEx research methodology. The panel is involved in interview guide development, sample selection strategies, identification of missing topics and characteristics in interview data as well as in helping with arising issues. The panel will also review the materials prepared for the PExMS-website.

PwMS are involved in content development of the intervention, as previously reported. Before uploading the multimedia files on the PExMS website, participants will be given transcriptions of the media and asked again for permission for uploading. The preliminary version of the PExMS website will be evaluated by pwMS. Dissemination of results to participants will be conducted via letters/newsletters and websites of MS self-help organisations.

### Adverse event reporting and harms

There is no guarantee that MS patients will personally benefit from this study. Studies investigating PEx as an intervention found no adverse events [14, 22, 35]. Since we do not expect relevant adverse events, no reporting is planned. Nevertheless, HADS as a control parameter will be applied to control for anxiety and depression.

### Plans for communicating important protocol amendments to relevant parties

Important protocol modifications will be communicated to the ethical committee, the advisory panel as well as to the trial registry and principal investigators.

### Dissemination plans

The results of our study will be presented at national and international conferences and meetings and published in peer-reviewed journals.

## Discussion

After diagnosis, pwMS are confronted with a variety of management options and have to make complex decisions such as deciding on immunotherapies. PEx as a supplement to factual information may support pwMS in decision-making [16–18, 23, 25, 28]. Using PEx is controversial because they appear to be randomly generated, situation-related, and possibly inaccurate with respect to scientific evidence and may bias decision-making [25, 29]. Therefore, systematic methods with active patient involvement are necessary to obtain and analyse the impact of PEx. This two-arm RCT will be carried out as an innovative pilot trial, since there are very few previously published controlled studies using PEx. The pilot trial will aid in designing a future RCT by providing knowledge on acceptability and quantification of feasibility in the form of recruitment and estimates of retention rates and the impact of the interventions on different outcome measures. The estimated effect size and confidence interval from the pilot trial may give some indication of whether the PExMS website might show effectiveness in immunotherapy decision-making processes in a larger trial [52, 53].

### Trial status

Trial registration: NCT04236544. Registered 22 January 2020. Prospectively registered, https://clinicaltrials.gov/ct2/show/NCT04236544. Recruitment has not yet begun.

## Supplementary Information

**Additional file 1.** SPIRIT 2013 Checklist: Recommended items to address in a clinical trial protocol and related documents.

## Data Availability

Data sharing is not applicable to this article as no datasets were generated or analysed during the current study.

## Abbreviations

CPS: Control Preferences Scale
DCS: Decisional Conflict Scale
DSES: Decision Self-Efficacy Scale
DIPEx: Database of Individual Patients’ Experience of illness
eHIQ: e-Health Impact Questionnaire
HADS: Hospital Anxiety and Depression Scale
MS: Multiple sclerosis
PDDS: Patient Determined Disease Steps
PEx: Patient experiences
PExMS: Patient Experiences of Multiple Sclerosis
PPMS: Primary-progressive multiple sclerosis
pwMS: Patients with multiple sclerosis
PrepDM: Preparation for decision-making
RCT: Randomised controlled trial
RIKNO: Risk Knowledge in Relapsing Multiple Sclerosis
RRMS: Relapsing-remitting multiple sclerosis
SPMS: Secondary-progressive multiple sclerosis
SURE: Sure of myself, Understand information, Risk-benefit ratio, Encouragement
